# A randomized controlled trial of surf and hike therapy for U.S. active duty service members with major depressive disorder

**DOI:** 10.1186/s12888-022-04452-7

**Published:** 2023-02-17

**Authors:** Kristen H. Walter, Nicholas P. Otis, Travis N. Ray, Lisa H. Glassman, Jessica L. Beltran, Kim T. Kobayashi Elliott, Betty Michalewicz-Kragh

**Affiliations:** 1 Health and Behavioral Sciences, Naval Health Research Center, 140 Sylvester Road, San Diego, CA 92106 USA; 2 Leidos, Inc., San Diego, CA USA; 3 Department of Public Health, Naval Medical Center San Diego, San Diego, CA USA

**Keywords:** Depression, Physical activity, Exercise, Nature exposure, Nature-based recreation therapy, Natural environment, Outdoor activity, Military, Randomized controlled trial

## Abstract

**Background:**

Major depressive disorder (MDD) is the most prevalent mental health disorder worldwide, including among U.S. service members. In addition to evidence-based treatments, activity-based approaches have been shown to effectively treat depressive symptoms, particularly when they occur in the natural environment.

**Methods:**

This study compared two activity-based interventions, Surf Therapy and Hike Therapy, on depression outcomes among 96 active duty service members with MDD. Participants were randomized to 6 weeks of Surf or Hike Therapy. Clinician-administered and self-report measures were completed at preprogram, postprogram, and 3-month follow-up. A brief depression/anxiety measure was completed before and after each activity session.

**Results:**

Multilevel modeling results showed that continuous depression outcomes changed significantly over time (*p*s < .001). Although service members in Hike Therapy reported higher average depression scores than those in Surf Therapy, the trajectory of symptom improvement did not significantly differ between groups. Regarding MDD diagnostic status, there were no significant differences between the groups at postprogram (*p* = .401), but Surf Therapy participants were more likely to remit from MDD than were those in Hike Therapy at the 3-month follow-up (*p* = .015).

**Limitations:**

The sample consisted of service members, so results may not generalize to other populations. Most participants received concurrent psychotherapy or pharmacotherapy, and, although statistically accounted for, results should be interpreted in this context.

**Conclusions:**

Both Surf and Hike Therapies appear to be effective adjunctive interventions for service members with MDD. Research is needed to examine the effectiveness of these therapies as standalone interventions.

**Trial registration:**

Clinical trials registration number

NCT03302611; First registered on 05/10/2017.

**Supplementary Information:**

The online version contains supplementary material available at 10.1186/s12888-022-04452-7.

## Introduction

Major depressive disorder (MDD) is a prevailing mental health disorder, including among U.S. service members [1, 2], with an estimated prevalence of 8% across branches [3]. MDD is associated with elevated rates of substance abuse, suicidal ideation, and other comorbidities [4–6]. In addition to mental health consequences, there are significant financial costs associated with MDD that can be attributed to reduced productivity [2], military strength, and operational readiness.

Fortunately, existing evidence-based treatments, such as cognitive behavioral therapy and antidepressant medications, are efficacious in treating MDD [7–10]. Antidepressant medications are also widely used for depression, with the largest benefits observed in patients with severe symptoms [11, 12] and in combination with psychotherapies [13]. However, among patients with MDD, 53 and 67% exhibit non-response and non-remission, respectively, to first-line treatment [14]. Antidepressants may not be desirable for all patients, and may produce side effects that lead to poor compliance and ineffective treatment [15, 16]. Further, once antidepressant use ends, there is no evidence of sustained treatment effects to prevent depression relapse [17, 18].

Given the variability in treatment tolerance and response [19, 20], there has been growing interest in evaluating adjunctive therapies for MDD. Various forms of physical activity can have pronounced effects on MDD and depressive symptoms [21–23]. Meta-analyses have suggested that exercise is a viable adjunctive intervention to standard treatment [24–27] and may be well tolerated for individuals with depression [28, 29].

The effectiveness of physical activity on improving mental health may be due to several factors. One factor, social interaction, is associated with improved psychological health [30] and alleviation of depressive symptoms [28]. In service members, the connections made during sports programs may provide normalization of shared experiences and motivation to improve relationships [31–34]. Additionally, physical activity that occurs in the natural environment has a larger effect on depressive symptoms than physical activity indoors [35]. Hiking/walking in nature has demonstrated greater improvements in depression [36, 37] and related symptoms [38, 39] compared with walking in an indoor or urban area. Exercise programs that incorporate social interaction and the natural environment may uniquely support the rehabilitation of mental health disorders [40].

Although hiking/walking is physical activity that can incorporate social interaction within a natural environment, a water-based environment or “blue space” [41] may confer greater benefits through unique sensory information created by the environment [42]. In support of this concept, Barton and Pretty (2010) [43] demonstrated that exercise outdoors near water generated greater mood improvements compared with exercise in a natural environment without water. Additionally, programs that encompass water-based activities within the natural environment have yielded psychological benefits for military veterans [44–46].

Surfing is a water-based activity shown to have beneficial effects for mental health in military samples. Among veterans and service members, surf therapy—an intervention using surfing to promote well-being (International Surf Therapy Organization, 2019; https://intlsurftherapy.org)—is associated with reduced depressive symptoms [47–49]. Furthermore, over the course of a surf therapy session, service members reported significant improvements in depression/anxiety, highlighting the immediate psychological benefits. These effects were enhanced among service members with probable MDD [49]. Collectively, these studies offer support for the use of surf therapy as an adjunctive intervention to improve symptoms of depression among military samples.

Despite preliminary evidence for the psychological benefits of surf therapy, there are limitations in the literature. Currently, few studies [31, 47–49] have examined the self-reported psychological effects of surf therapy among veterans and service members. There have been no studies utilizing clinician-administered measures for depression and associated symptoms. The duration of effects of surfing also remains unclear. There is some evidence that physical activity in the natural environment produces positive effects on mental health immediately following the activity [35, 49]. However, it is uncertain whether these psychological benefits are sustained over time. To date, Crawford (2016) [47] is the only study to report follow-up findings, which were limited to a 30-day period.

This study aims to address these limitations and evaluate the efficacy of activity-based interventions for MDD. Importantly, there are no published studies examining the effects of surf therapy using a randomized controlled trial (RCT) design or evaluating depressive symptoms following hike therapy in those with MDD. The rigorous design allows for the examination of both surf and hike therapy, providing clinical knowledge of intervention effects for service members with MDD. As a control condition, hiking is a sound comparison to surfing. Hiking is a physical activity with similar energy expenditure to surfing [50], allows for social interaction, and occurs in the natural environment. The primary aim for this study was to determine whether Surf Therapy produced greater reductions in depressive symptoms than Hike Therapy. We hypothesized that both therapies would significantly decrease MDD symptoms and remission rates during the study period; however, we expected Surf Therapy to show greater effects compared with Hike Therapy, consistent with the blue space framework. Comparison of dropout rates and program satisfaction among participants in Surf and Hike Therapy was an exploratory aim. Results could ultimately serve to inform care provided to service members, including whether surf and hike therapies can augment standard treatment for MDD.

## Methods

### Participants

**Fig. 1:**
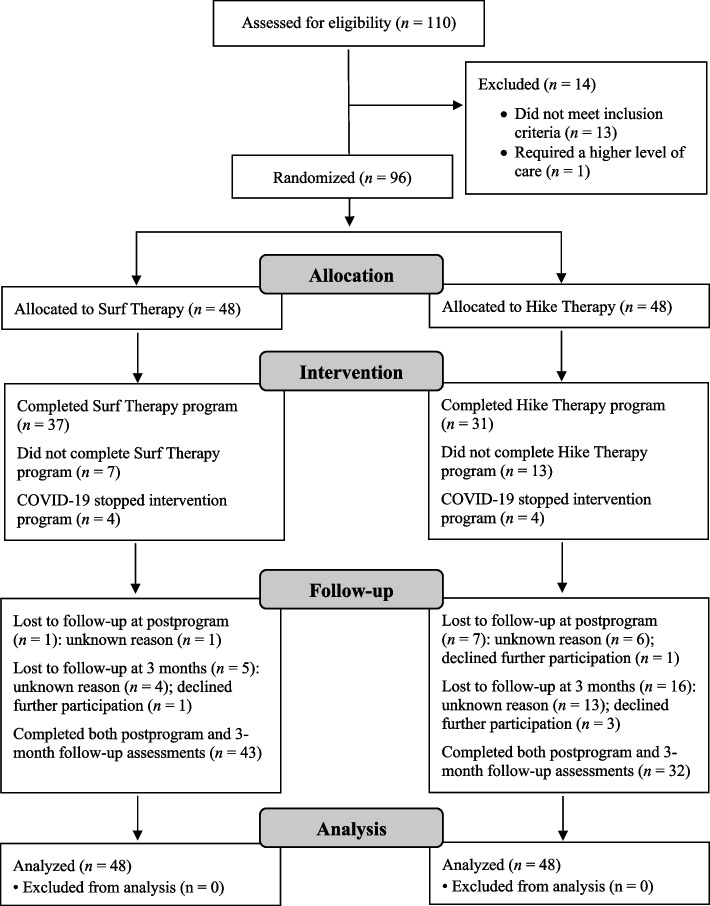
CONSORT flowchart of participants. Intervention “completion,” as determined by the NMCSD Surf and Hike Therapy programs, was defined as completing all but two of the available sessions (up to six sessions) for each modality. The programs were halted due to the COVID-19 pandemic; participants in this cohort (*n* = 8) were counted neither as completers nor non-completers. Their data were analyzed as intent to treat. Total lost to follow-up is greater than the total number allocated because many participants completed *either* the postprogram assessment *or* the follow-up assessment and are thus counted twice. RCT = randomized controlled trial; COVID-19 = coronavirus disease 2019

Participants included 110 active duty service members referred to the Wounded, Ill, and Injured (WII) Wellness Program at Naval Medical Center San Diego (NMCSD) between January 2018 and March 2020 (see CONSORT diagram; Fig. 1). Service members were eligible for participation if they met diagnostic criteria for MDD as assessed by the Mini International Neuropsychiatric Interview 7.0 (MINI-7) [51]. The only exclusion criterion was previous participation in the Surf or Hike Therapy programs, to control for intervention dose. All participants received medical clearance from providers at the Naval Hospital prior to participation in the Surf and Hike Therapy programs. Power calculations have been previously published (see ﻿﻿﻿[52]﻿)﻿﻿. Eligible participants were randomly assigned to either Surf or Hike Therapy, using a blocked randomization design (http://randomization.com; 11 blocks of 10 participants each) to balance treatment groups throughout recruitment. Participants who were engaged in psychotherapy or prescribed psychotropic medication were not excluded from the study, but concurrent treatment data were collected. Participants were permitted to engage in the non-randomized intervention during the follow-up period given the transitory nature of military service. Written informed consent was obtained from all participants involved in the study, and participation was voluntary.

### Program

The Surf and Hike Therapy programs are provided as an option of standard care at NMCSD. Both programs consisted of six consecutive weekly sessions, each lasting 3 to 4 hours. In this study, the programs used a cohort format accommodating approximately 20 service members per cycle. The Surf Therapy program occurred at a public beach in San Diego and the Hike Therapy Program took place at various locations throughout San Diego County. Optional yoga was offered to Surf Therapy participants before each session as part of the existing NMCSD program. Information about the Surf and Hike Therapy programs has been published elsewhere [52]. Given the sudden onset and widespread impact of the COVID-19 pandemic, the WII Wellness Program canceled in-person group activities; the last cohort of participants were unable to complete their respective programs that began in March 2020, and study enrollment ended; however, adequate power for analysis was achieved.

### Procedure

Participants were assessed at preprogram, postprogram, 3-month follow-up, and within each session. All participants completed a preprogram assessment that included clinical interview and self-report measures evaluating MDD and related symptoms, after which they were assigned a randomized condition if eligible for study inclusion. The study assessor was blind to participants’ randomized condition throughout the study. Within 2 weeks of the preprogram assessment, participants began their assigned program. During the 6-week programs, participants completed brief self-report assessments immediately prior to and following each session. Within 2 weeks after their final weekly session, participants completed a postprogram assessment, and a final assessment 3 months later. Participants did not receive financial compensation but were allowed to keep the Fitbit device used for secondary data collection if the device was worn for at least 50% of the study period. Study procedures were approved by the Naval Medical Center San Diego Institutional Review Board. Study methods were performed in compliance with all applicable Federal regulations governing the protection of human subjects.

### Measures

Preprogram measures included participant demographics, service characteristics, concurrent treatment utilization (i.e., psychotherapy or pharmacotherapy for depression), depressive symptoms, and physical activity. Preprogram physical activity was assessed with the 7-item International Physical Activity Questionnaire-Short Form (IPAQ-SF) [53]. The IPAQ-SF is summed according to the IPAQ Manual [54] and reflects the frequency and intensity of physical activity over the last 7 days in metabolic equivalent minutes (MET mins) via three categories: low (< 600 MET mins/week), medium (600–2999 MET mins/week), and high (≥3000 MET mins/week).

#### Depression measures

The primary outcome measure was the Montgomery–Åsberg Depression Rating Scale (MADRS) [55], a clinician-rated measure used to evaluate depression severity. The MADRS consists of 10 items rated from 0 to 6, with higher scores reflecting greater depression severity. Internal consistency for the MADRS ranged from α = 0.74 to 0.90 across time points. The MINI-7 [51] is a clinician-administered interview and used to assess MDD diagnostic criteria. The MADRS and MINI-7 were administered at preprogram, postprogram, and 3-month follow-up.

Self-reported depression severity was assessed with the 9-item Patient Health Questionnaire (PHQ-9) [56] at preprogram, postprogram, and 3-month follow-up. PHQ-9 items were rated from 0 to 3 and summed to yield a total severity score. Higher scores reflect greater depression symptom severity. Internal consistency in this study ranged from α = 0.77 to α = 0.89. The 4-item Patient Health Questionnaire (PHQ-4) [57] was used to assess symptoms of depression and anxiety before and after each session. Items were scored from 0 to 3 and summed to create a severity score, with higher scores indicating greater symptom severity. Internal consistency ranged from α = 0.77 to α = 0.89.

#### Participant satisfaction

Participant satisfaction with each program was assessed with the 8-item Client Satisfaction Questionnaire (CSQ-8) [58]. Items were rated from 1 to 4 and summed to a total satisfaction score, with higher scores suggesting greater satisfaction. Internal consistency for the CSQ-8 was α = 0.88.

### Assessment reliability

The MINI and MADRS were audio-recorded across time points. Twenty percent of participants (*n* = 22) were randomly selected for review by a second rater to determine assessment reliability. The reliability assessor reviewed all available time points for each participant selected. The reliability assessor holds a doctoral degree in clinical psychology and has expertise in the diagnosis of MDD and extensive experience using both instruments. Intraclass correlations (ICCs) were excellent for the MINI MDD module (ICC = .91) and MADRS (ICC = .91).

### Data analysis

Data were analyzed as intent-to-treat; therefore, all service members eligible for study participation (*N* = 96) were included in the analyses. Multilevel modeling (MLM) was used to examine depression symptom changes over time (longitudinally and within session) for both conditions. This analytic approach accounts for missing data and correlated error due to repeated measures and across time while nested within groups. MDD remission rates at postprogram and 3-month follow-up were compared using chi-square tests of association and McNemar’s test. Cohen’s *g* [59] was used to calculate the effect sizes of these remission rates. An independent samples *t* test was used to compare participant satisfaction at postprogram. Using chi-square tests of association and independent samples *t* tests, dropout analyses examined differences between completers and non-completers. Participants who elected to repeat the program to which they were randomized or who participated in the other program during their follow-up period were coded accordingly, and these variables were used in analyses. Analyses were conducted using IBM SPSS Statistics software version 25 (IBM, Armonk, NY).

Both longitudinal (preprogram, postprogram, 3-month follow-up) and within session (presession, postsession) analyses were conducted with MLM using a step-up model building process. Logical covariance matrices were compared and selected based on model fit according to Akaike information criterion with respect to the number of parameters specified. All final multilevel models used restricted maximum likelihood to account for missing data.

For longitudinal analyses, the intercept was set as a random effect of subject with a diagonal covariance matrix. Time was a repeated effect with a covariance matrix of scaled identity. Piecewise analysis was used with longitudinal MLM models to best account for different independent variables in the intervention and follow-up periods (e.g., number of sessions attended in each time frame). Fixed effects were specified as follows: time (0 = preprogram, 1 = postprogram in pre- to post-models; 0 = postprogram, 1 = 3-month follow-up in follow-up models); intervention condition (0 = Hike Therapy, 1 = Surf Therapy); concurrent pharmacotherapy for depression (0 = no, 1 = yes); concurrent psychotherapy for depression (0 = no, 1 = yes); physical activity level (0 = high, 1 = moderate, 2 = low); and number of exercise therapy sessions attended (continuous total). Each fixed effect was also used in an interaction term with time.

For within-session analysis, intercept, time (pre- to postsession), week of session, and a crossed effect of Time × Week of Session were set as random slopes by subject with a first-order autoregressive covariance matrix. Time × Week of Session was set as a repeated effect of subject and used a compound symmetry covariance matrix. Fixed effects included time (0 = presession, 1 = postsession); intervention condition (0 = Hike Therapy, 1 = Surf Therapy); concurrent pharmacotherapy for depression (0 = no, 1 = yes); concurrent psychotherapy for depression (0 = no, 1 = yes); physical activity level (0 = high, 1 = moderate, 2 = low); and week of exercise session (continuous week number). All fixed effects were also used in individual interactions with time.

## Results

Study sample characteristics are displayed in Table 1. Of the 96 eligible participants, 48 were randomized to each Surf and Hike Therapy. Age (*M*_diff_ = − 2.3, *p* = .045) and preprogram PHQ-9 scores (*M*_diff_ = 2.2, *p* = .025) significantly differed by intervention group; Hike Therapy participants were younger and self-reported higher depression scores. Otherwise, no other variables significantly differed between groups. Of the overall sample, 77.3% were intervention completers, defined by the programs as missing no more than two sessions. Neither completion rates (*p* = .127) nor average number of sessions attended (*p* = .656) significantly differed by intervention group. Regarding assessment, 91.7% of participants completed at least one follow-up assessment, and 78.1% completed both. Among those who participated in a second round of intervention during the follow-up period (*n* = 38), 28 repeated their randomized intervention (25 surf; 3 hike) and 10 selected their non-randomized intervention (8 switched to surf; 2 switched to hike). Of those who completed a second intervention round, the average number of sessions attended was 3.3 (SD = 1.7). Program satisfaction was high (*M* = 29.3, SD = 3.5) and there was no significant difference by intervention group (*p* = .070). During the study period, there were two adverse events—both of which occurred in the Surf Therapy group. One was an expected and non-severe injury sustained during the activity, and another was unrelated to study procedures.

**Table 1:** Preprogram sample characteristics

Characteristic	Total sample (*N* = 96)	Surf (*n* = 48)	Hike (*n* = 48)
	*n* (%)	*n* (%)	*n* (%)
Race/ethnicity^a^			
White	40 (41.7)	–	–
Multiracial	19 (19.8)	–	–
Hispanic, Latinx, or Spanish origin	18 (18.8)	–	–
Black or African-American	15 (15.6)	–	–
Asian or Asian-American/Native American or Alaska Native	4 (4.2)	–	–
Gender identity^b^			
Female	50 (52.1)	22 (45.8)	28 (58.3)
Male	46 (47.9)	26 (54.2)	20 (41.7)
Rank^a^			
E1–E4	34 (35.4)	–	–
E5–E9	57 (59.4)	–	–
Officer	5 (5.2)	–	–
Concurrent depression treatment^c^	87 (90.6)	43 (89.6)	44 (91.7)
Pharmacotherapy	63 (65.6)	32 (66.7)	31 (64.6)
Psychotherapy	86 (89.6)	42 (87.5)	44 (91.7)
Activity level^d^			
Low/inactive	10 (10.4)	8 (16.7)	2 (4.2)
Moderately active	37 (38.5)	18 (37.5)	19 (39.6)
Highly active	32 (33.3)	13 (27.1)	19 (39.6)
Completion of assigned program^e,f^	68 (77.3)	37 (84.1)	31 (70.5)
	M (SD)	M (SD)	M (SD)
Age, years	28.1 (5.6)	29.3 (6.2)*	27.0 (4.8)*
Education, years	13.2 (1.7)	13.3 (1.7)	13.2 (1.7)
Sessions attended^f,g^	3.9 (1.6)	3.9 (1.3)	4.0 (1.9)
Yoga sessions attended	0.7 (1.2)	1.3 (1.4)	–
Preprogram measures			
MADRS	27.0 (8.4)	25.9 (8.2)	28.0 (8.6)
PHQ-9	17.07 (4.9)	16.0 (4.8)*	18.2 (4.8)*

*E* Enlisted rank, *MADRS* Montgomery-Åsberg Depression Ranking Scale, *PHQ-9* 9-item Patient Health Questionnaire. Asterisks indicate significant difference between activity conditions. Totals may not sum to sample numbers or percentages due to missing data

^a^All attempts were made to report race/ethnicity and rank data properly, but due to low cell counts, variables were combined to protect participant identity, and they are not stratified by condition

^b^Gender identity was self-reported

^c^Because concurrent depression treatment categories are not mutually exclusive, the sum across treatment categories is greater than 100%

^d^Physical activity level data were calculated (see IPAQ Research Committee, 2005) from the self-report version of the IPAQ-SF

^e^Program completion was defined by the NMCSD Surf and Hike Therapy Programs as missing no more than two sessions of the assigned modality

^f^Because programs were halted due to the sudden onset of COVID-19, participants (*n* = 8) in the affected cohort were not counted in completion or attendance statistics

^g^Included are only sessions in which the assigned modality was conducted. Occasionally, due to adverse weather, sessions consisted of alternative activities (e.g., visit to the National Surf Museum)

*p < .05; **p < .01

### Piecewise longitudinal analyses

Piecewise longitudinal analyses were conducted from pre- to postprogram and again from postprogram to 3-month follow-up. Means and standard deviations for outcome variables are in Appendix A. Base models for both MADRS and PHQ-9 revealed significant effects of time from pre- to postprogram (*p*s < .001). For base models examining postprogram to 3-month follow-up, time was statistically significant for both MADRS and PHQ-9 (*p*s <. 001). Final models for pre- to postprogram and postprogram to 3-month follow-up analyses examining MADRS and PHQ-9 included time, intervention condition, concurrent pharmacotherapy, concurrent psychotherapy, the number of sessions attended in the respective time period, physical activity levels at preprogram, and their interactions with time.

#### Final MADRS models

**Fig. 2:**
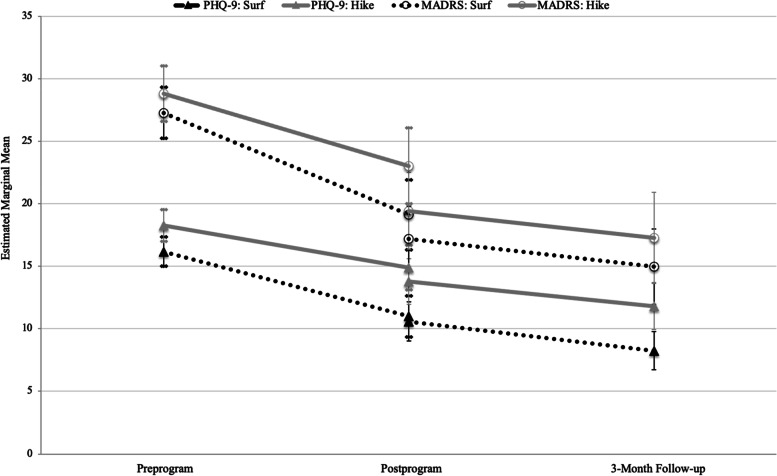
Estimated marginal means of MADRS and PHQ-9 at study assessment time points. Graph line is discontinuous due to separate piecewise multilevel model analyses, which used independent variables relative to each time point. MADRS = Montgomery-Åsberg Depression Rating Scale; PHQ-9 = 9-item Patient Health Questionnaire

Final MLM results for the MADRS are featured in Table 2, and a graph of estimated marginal means in Fig. 2. In the final pre- to postprogram model, the effect of time was significant for MADRS (B = − 8.49 *p* < .001). Averaged across participants and adjusted for other predictors, MADRS scores decreased from 27.45 to 18.96 from pre- to postprogram, representing both clinical and statistical significance. The only significant main effect was the number of sessions attended (B = − 1.87, *p* = .024), where those who attended more sessions had lower average MADRS scores across time. Otherwise, no other significant main effects of independent variables were evident (*p*s = .183–.997). A significant interaction was found for Time × Sessions Attended (B = − 1.56, *p* = .043), indicating that larger improvement in MADRS score was related to a greater number of sessions attended. There were no significant effects of concurrent enrollment in pharmacotherapies (*p* = .076) or psychotherapies (*p* = .450) for depression, nor were there any effects of baseline physical activity on trajectories from pre- to postprogram (*p*s = .591–.932). There was no difference in MADRS scores’ change over time by intervention condition (*p* = .376); participants in both Surf and Hike Therapy showed significant reductions in depression symptom severity.

**Table 2:** Estimates of fixed effects of final multilevel models examining MADRS over time

	Pre- to postprogram			Postprogram to 3-month follow-up		
Variable	B	95% CI	*p*	B	95% CI	*p*
Intercept	27.44	[21.58, 33.31]	**<.001**	21.21	[13.14, 29.28]	**<.001**
Time	−8.49	[− 10.60, − 6.37]	**<.001**	− 3.80	[−4.47, − 3.13]	**<.001**
Intervention condition	−3.92	[−9.74, 1.90]	.183	−2.30	[−9.31, 4.71]	.515
Time × Intervention Condition	2.41	[−2.98, 7.80]	.376	0.07	[− 5.41, 5.55]	.979
Pharmacotherapy	−1.65	[−8.30, 5.00]	.622	0.68	[−6.78, 8.13]	.857
Time × Pharmacotherapy	5.56	[−0.61, 11.73]	.076	0.94	[−4.93, 6.82]	.749
Psychotherapy	−0.02	[−10.28, 10.25]	.997	8.61	[−1.81, 19.04]	.104
Time × Psychotherapy	−3.60	[−13.08, 5.87]	.450	−2.97	[−11.34, 5.40]	.480
Activity level^a^	–	–	–	–	–	–
Moderate	2.45	[−6.59, 11.49]	.591	0.87	[−8.84, 10.57]	.859
High	−0.38	[−9.37, 8.60]	.932	−2.27	[−12.17, 7.62]	.647
Time ×Activity Level^a^	–	–	–	–	–	–
Time × Moderate	−0.40	[−8.74, 7.93]	.924	2.43	[−4.93, 9.78]	.511
Time × High	−1.57	[−9.83, 6.68]	.705	2.98	[−4.55, 10.52]	.431
Sessions attended	−1.87	[−3.48, −0.25]	**.024**	−0.93	[−2.62, 0.76]	.276
Time × Sessions Attended	−1.56	[−3.06, −0.05]	**.043**	−0.42	[−1.72, 0.88]	.518

*MADRS* Montgomery-Åsberg Depression Rating Scale. Significant values are bolded

Pharmacotherapy and psychotherapy use is relative to each time point and was used as a predictor; for pre- to postprogram, preprogram usage was utilized; for postprogram to follow-up, postprogram usage was utilized. Similarly, the sessions attended variable represents those from pre- to postprogram and postprogram to follow-up, respectively, if applicable

^a^Groupings according to standard IPAQ-SF scoring: low (< 600 MET mins/week), medium (600–3000 MET mins/week), high (> 3000 MET mins/week). Here, the Low group is the referent category because this group does not meet the World Health Organization guidelines for physical activity

In the final postprogram to 3-month follow-up MADRS model, time was significant (B = − 3.79, *p* < .001), indicating that scores changed from postprogram to 3-month follow-up. All other independent variables showed nonsignificant main effects (*p*s = .104–.857) and interactions (*ps* = .431–.979). Taken together, participants generally showed reduced depression severity on the MADRS in the follow-up period, and that symptom change did not vary due to intervention condition, concurrent pharmacotherapy, concurrent psychotherapy, the number of sessions attended during the follow-up period, or preprogram physical activity levels.

#### Final PHQ-9 models

Final MLM results for the PHQ-9 are shown in Table 3, and a graph of estimated marginal means in Fig. 2. Like MADRS models, the effect of time from pre- to postprogram was significant for PHQ-9 scores (B = − 4.88, *p* = < .001). Average PHQ-9 scores decreased from 16.67 to 11.78 (moderately severe to moderate level) across participants, indicating both statistically and clinically significant improvement from pre- to postprogram. Although those in Surf Therapy reported lower preprogram PHQ-9 scores than those in Hike Therapy (B = − 3.91, *p* = .026), there was no significant difference between groups for improvement over time (*p* = .254). There were also no significant main effects of pharmacotherapy, psychotherapy, or preprogram physical activity (*p*s = .399–.931). Interaction effects showed that participants who received concurrent pharmacotherapy for depression improved more from pre- to postprogram (B = 3.65, *p* = .047) compared with those who did not, while psychotherapy did not demonstrate a significant interaction with time (*p* = .468). Preprogram physical activity level did not significantly impact the average trajectory from pre- to postprogram (*ps* = .479–.511). Lastly, while those who attended more sessions had lower PHQ-9 scores at each time point (B = − 0.99, *p* = .041), the number of sessions attended did not significantly impact the change in PHQ-9 scores (*p* = .191).

**Table 3:** Estimates of fixed effects of final multilevel models examining PHQ-9 over time

	Pre- to postprogram			Postprogram to 3-month follow-up		
Variable	B	95% CI	*p*	B	95% CI	*p*
Intercept	16.67	[13.33, 20.01]	**<.001**	13.06	[8.22, 17.89]	**<.001**
Time	−4.88	[−6.24, − 3.53]	**<.001**	−2.54	[−2.90, − 2.19]	**<.001**
Intervention condition	− 3.91	[−7.33, − 0.49]	**.026**	−3.56	[− 7.16, 0.03]	.052
Time × Intervention Condition	1.81	[−1.33, 4.96]	.254	0.37	[−2.58, 3.31]	.805
Pharmacotherapy	−1.03	[−4.94, 2.87]	.599	−1.28	[−5.10, 2.54]	.505
Time × Pharmacotherapy	3.65	[0.05, 7.25]	**.047**	1.88	[−1.27, 5.02]	.237
Psychotherapy	0.26	[−5.77, 6.30]	.931	4.83	[−0.51, 10.16]	.075
Time × Psychotherapy	−2.02	[−7.55, 3.51]	.468	−3.24	[−7.69, 1.21]	.150
Activity level^a^	–	–	–	–	–	–
Moderate	2.26	[−3.06, 7.57]	.399	2.08	[−2.90, 7.06]	.407
High	1.29	[−3.99, 6.58]	.626	0.81	[−4.27, 5.89]	.751
Time x Activity Level^a^	–	–	–	–	–	–
Time × Moderate	−1.61	[−6.47, 3.25]	.511	0.90	[−3.08, 4.87]	.653
Time × High	−1.72	[−6.54, 3.10]	.479	1.02	[−3.06, 5.10]	.618
Sessions attended	−0.99	[−1.94, −0.04]	**.041**	−0.24	[−1.11, 0.62]	.574
Time × Sessions Attended	−0.58	[−1.46, 0.30]	.191	−0.13	[−0.83, 0.57]	.702

*PHQ-9* 9-item Patient Health Questionnaire. Significant values are bolded

Pharmacotherapy and psychotherapy use is relative to each time point and used as a predictor; for pre- to postprogram, preprogram usage was utilized; for postprogram to follow-up, postprogram usage was utilized. Similarly, the sessions attended variable represents those from pre- to postprogram and postprogram to follow-up, respectively, if applicable

^a^Groupings according to standard IPAQ-SF scoring: low (< 600 MET mins/week), medium (600–3000 MET mins/week), high (> 3000 MET mins/week). Here, the Low group is the referent category because this group does not meet the World Health Organization guidelines for physical activity

In final postprogram to 3-month follow-up PHQ-9 models, the main effect of time was significant (B = − 2.54, *p* = < .001). In the follow-up period, there were no significant differences in self-reported depression severity (*p* = .052) or rate of symptom improvement between participants in Surf and Hike Therapy (*p* = .805). All other predictor variables and their interactions with time were nonsignificant (*p*s = .075–.751), indicating that self-reported depression severity was not significantly impacted by concurrent treatments, preprogram physical activity level, or the number of sessions attended during the follow-up period.

### Diagnostic outcomes

At postprogram, participants in both interventions showed significant within-group changes in rates of MDD diagnosis using McNemar’s test (*p*s < .001; Surf = 55%; Hike = 46% without MDD), and these rates did not significantly differ between groups using Pearson’s chi-squared test (*p* = .401). Effect sizes for this pre-to-postprogram diagnostic change were large for both groups (Cohen’s *g*’s = 0.50). At the 3-month follow-up, participants in Surf (*p* = .092) and Hike (*p* = 1.00) Therapy did not show significant within-group change from postprogram to 3-month follow-up. However, a significant difference emerged between the intervention groups (*p* = .015): participants in Surf Therapy (74% without MDD) showed greater rates of MDD remission than those in Hike Therapy (47% without MDD). The effect sizes for these diagnostic changes from postprogram to 3-month follow-up were large for the Surf Therapy group (Cohen’s *g* = 0.27) and negligible for the Hike Therapy group (Cohen’s *g* = 0.0).

### Within-session analyses

Means and standard deviations for PHQ-4 scores can be found in Appendix A. An initial base model showed a significant effect of time (*p* < .001) on brief, self-reported depression/anxiety scores. Yoga attendance was not included in final models because only 28% of the sample participated and only those in Surf Therapy due to existing programming. Final models included time, intervention group, pharmacotherapy, psychotherapy, week of session, and their interactions.

The final pre- to postsession model for the PHQ-4 is shown in Table 4. The main effect of time was significant (B = − 3.22, *p* < .001), demonstrating that on average, across participants, depression/anxiety improved over the course of a session. A significant effect of intervention condition (B = − 1.35, *p* = .018), coupled with a nonsignificant interaction of Session × Intervention Condition (*p* = .217), indicated that although those in Hike Therapy began each session with higher PHQ-4 scores relative to those in Surf Therapy, the degree of improvements over each session did not differ by group. A nonsignificant Session × Week Interaction (*p* = .136) showed that the amount of change within each session was consistent across weekly sessions. Additionally, there were no main or interaction effects of concurrent pharmacotherapy (*p* = .490 and .254, respectively) or psychotherapy for depression (*p* = .302 and .297, respectively) on PHQ-4 scores from pre- to postsession. This suggests that neither type of concurrent treatment impacted depression/anxiety scores over the course of a session. Main effects (*p*s = .174–.589) and interactions (*p*s = .285–.322) were also nonsignificant for preprogram physical activity levels.

**Table 4:** Estimates of fixed effects of final multilevel models examining PHQ-4 over time

Variable	B	95% CI	*p*
Intercept	6.54	[4.88, 8.20]	**<.001**
Time (pre- to postsession)	−3.22	[−3.22, − 3.21]	**<.001**
Intervention condition	− 1.35	[−2.47, 0.24]	**.018**
Time × Intervention Condition	0.46	[− 0.27, 1.20]	.217
Pharmacotherapy	0.45	[−0.85, 1.75]	.490
Time × Pharmacotherapy	0.49	[−0.36, 1.34]	.254
Psychotherapy	−0.99	[−2.90, 0.91]	.302
Time × Psychotherapy	0.66	[−0.59, 1.92]	.297
Activity level^a^	–	–	–
Moderate	1.26	[−0.57, 3.10]	.174
High	0.52	[−1.38, 2.41]	.589
Time x Activity Level^a^	–	–	–
Time × Moderate	−0.63	[−1.87, 0.62]	.322
Time × High	−0.70	[−1.98, 0.59]	.285
Week of session	−0.05	[−0.23, 0.13]	.558
Time × Sessions Attended	0.17	[−0.05, 0.39]	.136

*PHQ-4* 4-item Patient Health Questionnaire. Significant values are bolded

^a^Groupings according to standard IPAQ-SF scoring: low (< 600 MET mins/week), medium (600–3000 MET mins/week), high (> 3000 MET mins/week). Here, the Low group is the referent category because this group does not meet the World Health Organization guidelines for physical activity

## Discussion

The growing interest in exercise and adjunctive interventions for MDD calls for rigorous evaluation to determine their efficacy. Surf therapy and blue space have received theoretical and practical consideration as an adjunctive intervention for psychological symptoms in military populations [31, 47–49]. The current study provided support for surf and hike therapies as effective adjunctive interventions for service members with MDD, and participants were highly satisfied with both programs. Results indicated that there were significant decreases in clinician-rated and self-reported depressive symptoms across groups. The reductions in clinician-rated depressive symptoms were large, clinically significant, and greater among service members who attended more sessions. The programs were also associated with statistically and clinically significant improvements in self-reported depressive symptoms, and effects were stronger among those enrolled in pharmacotherapy. Regarding MDD remission, there were significant within-group changes for both intervention groups from pre- to postprogram but not in the follow-up period. The intervention groups significantly differed on MDD remission at the 3-month follow-up; Surf Therapy participants were more likely to remit from an MDD diagnosis compared to those in Hike Therapy. Importantly, results showed that for both interventions, gains were maintained and continued to improve in the follow-up period.

In addition to the lasting effects of Surf and Hike Therapy, findings supported immediate effects on depression/anxiety symptoms. Specifically, there were significant within-session improvements in depression/anxiety for both interventions. The pattern of results mirrored the changes documented in prior surf therapy research [49, 60], wherein symptoms largely decreased following each session but returned before the next session. This suggests that for weekly activities to have an effect, participants must receive a “dose” of surf or hike therapy. Although within-session findings indicated that there was no difference in the amount of depression/anxiety change each session throughout the weeks, pre- to postprogram results indicated that the change in clinician-rated depressive symptoms was reliant on the number of sessions that the participant attended. These results suggest that surf and hike therapy provide immediate psychological benefits, but a sufficient “dose” may be necessary for participants to maintain gains.

Contrary to expectations, there was little evidence for the enhanced effects of Surf Therapy relative to Hike Therapy. Although the Surf Therapy group reported less severe depressive symptoms during the intervention period, the trajectories of symptom change were parallel between the intervention groups across time, as well as within session. The only significant between-group difference was in MDD remission at the 3-month follow-up, for which service members in Surf Therapy (74% without MDD) were more likely to achieve MDD remission compared with those in Hike Therapy (47% without MDD). However, neither condition showed significant within-group changes from postprogram to 3-month follow-up. It should be noted that participants in Hike Therapy had more missing data at the 3-month follow-up, which may have affected the MDD remission rates between the conditions at this time point.

The mixed support for the blue space framework is reflected in research [43, 46]. Perhaps the effects of water are too subtle to emerge beyond the “statistical noise” when comparing symptoms across different activities (e.g., surfing/hiking) yet possess the strength to appear when the comparison activity is the same (e.g., walking in different settings). Discovering the conditions under which water-based effects emerge is an intriguing research challenge, but the comparable effects of hike and surf therapy on depressive symptoms are encouraging from a care provision standpoint. Hiking is a more accessible activity that has the potential to provide psychological benefits to a wider range of people, and not only to those who have the means to purchase equipment and live in proximity to a large body of water (e.g., [61, 62]).

Equally possible is that surf and hike therapies produced similar outcomes because they both align with clinical theories and approaches shown to effectively treat depression. For example, surf and hike therapies could be considered as forms of behavioral activation [63], an effective treatment for depression [64] that focuses on increasing an individual’s engagement in enjoyable activities and positive connection with the environment. Similarly, study findings highlight the theoretical underpinnings of cognitive behavioral therapy, an established psychotherapy [65] that involves changing behaviors to facilitate corrective experiences and challenge dysfunctional beliefs commonly experienced in depression [66, 67]. Stated differently, through engaging in surf or hike therapy, individuals may learn new skills (such as the activity itself or as a means of coping/relaxation), develop friendships, or build mastery, which can reduce depressive cognitions, behaviors, and symptoms. Surf and hike therapies may rest on foundational theoretical and psychotherapeutic approaches for depression, just through prescribed, group-based physical-activity in the natural environment.

Study results should be interpreted within the context of its limitations. The sample consisted of service members, so results may not generalize to other populations. Most participants self-reported at least moderate engagement in physical activity at preprogram; individuals who regularly engage in physical activity may be likely to choose and benefit from activity-based approaches [62]. Although hiking was a fair comparison to surfing in several ways, there may be inherent differences in the activities, such as skill required or environmental conditions, that may differentially affect psychological outcomes. Importantly, the study was conducted in accordance with WII Wellness program policies. Despite the strong external validity provided by keeping these policies intact, these policies also limit our ability to isolate effects. Concurrent treatment was permitted by our inclusion criteria for ethical reasons, and although we statistically controlled for shared variance, we were unable to fully detach the effects of the activity interventions from those of traditional treatments. Future research that includes a traditional treatment group without an activity-based intervention would help elucidate the variance accounted for by various treatment modalities. Lastly, because we compared our randomized conditions at the program level, we are unable to determine which factors (e.g., socializing, activity, nature) were most beneficial, or the extent to which these components varied by program.

This research also has strengths that advance the fields of surf therapy and activity-based intervention research. Notably, this study implemented an RCT design with gold standard clinician-administered measures. The RCT included an active comparison group to determine whether there were unique effects of water-based activities on symptoms of MDD. Further, all participants met diagnostic criteria for MDD, allowing outcomes to be evaluated within a clinical population for whom they may be particularly beneficial [49, 59, 68]. Data were collected on concurrent treatment variables, which were included in statistical models. This longitudinal study included a 3-month follow-up, providing greater granularity about the duration of effects. Overall, this study improved upon methodological limitations of prior activity-based intervention research, and findings offer greater confidence in these interventions as effective adjunctive approaches for service members with MDD.

## Conclusions

Activity-based therapies provide individuals with the opportunity to exercise, socialize, engage with the natural environment, and experience respite from their psychological symptoms [31, 32, 34, 69]. Study results suggest that surf and hike therapies can facilitate recovery from MDD among service members. Clinician-administered and self-reported depressive symptoms significantly improved within sessions and up to 3 months following program completion. Substantial rates of MDD remission were demonstrated, wherein over half of the participants no longer met the clinical threshold for MDD 3 months after the program concluded. Given that most participants were enrolled in traditional treatments, the results must be considered in this context. Surf and hike therapies, therefore, appear to be effective adjunctive treatments for service members with MDD, and may share principles consistent with cognitive behavioral therapies. These results are promising and contribute to the emerging evidence base supporting the use of activity-based interventions to address depressive symptoms and enhance psychological well-being. Additional research on surf and hike therapies is needed to inform whether these interventions are best as adjunctive or standalone treatments and under what circumstances, which will aid clinicians in effectively adapting treatment plans using these interventions.

## Supplementary Information

**Additional file 1: Table A1.** Means and standard deviations of depression outcomes at study assessment time points. **Fig. A1.** Raw average depression symptom scores at study assessment time points. MADRS = Montgomery-Åsberg Depression Rating Scale; PHQ-9 = 9-item Patient Health Questionnaire. **Table A2.** Means and standard deviations of depression/anxiety symptoms at session assessment time points.

## Data Availability

The protocol and datasets generated and/or analyzed during the current study are not publicly available due to security protocols and privacy regulations, but they may be made available on reasonable request by the Naval Medical Center San Diego or Naval Health Research Center IRBs (contact phone + 1.619.553.8400) or by contacting the corresponding author to facilitate the request.

## Abbreviations

CSQ-8: 8-Item Client Satisfaction Questionnaire
ICC: Intraclass correlation coefficient
IPAQ-SF: International Physical Activity Questionnaire – Short Form
MADRS: Montgomery-Åsberg Depression Response Scale
MDD: Major depressive disorder
MET: Metabolic Equivalent
MLM: Multilevel model
NMCSD: Naval Medical Center San Diego
PHQ-4: 4-Item Patient Health Questionnaire
PHQ-9: 9-Item Patient Health Questionnaire
RCT: Randomized controlled trial
