# Supplementary material for: A randomized controlled trial of surf and hike therapy for U.S. active duty service members with major depressive disorder
Source: BMC Psychiatry. 2023 Feb 17;23:109. doi: 10.1186/s12888-022-04452-7 (PMC9936467; doi:10.1186/s12888-022-04452-7)
Supplement: Supplementary file 1 — Additional file 1: Table A1. Means and standard deviations of depression outcomes at study assessment time points. Fig. A1. Raw average depression symptom scores at study assessment time points. MADRS = Montgomery-Åsberg Depression Rating Scale; PHQ-9 = 9-item Patient Health Questionnaire. Table A2. Means and standard deviations of depression/anxiety symptoms at session assessment time points. [file 12888_2022_4452_MOESM1_ESM.docx]

**Appendix A**

**Table A1**

Means and standard deviations of depression outcomes at study assessment timepoints.

|  | MADRS | | | PHQ-9 | | |
| --- | --- | --- | --- | --- | --- | --- |
| Timepoint | *n* | *M* | *SD* | *n* | *M* | *SD* |
| Preprogram | 95 | 26.96 | 8.42 | 96 | 17.07 | 4.89 |
| Surf | 48 | 25.92 | 8.23 | 48 | 15.96 | 4.78 |
| Hike | 47 | 28.02 | 8.56 | 48 | 18.19 | 4.78 |
| Postprogram | 88 | 19.94 | 11.41 | 88 | 12.08 | 6.90 |
| Surf | 47 | 18.62 | 11.35 | 47 | 10.72 | 6.61 |
| Hike | 41 | 21.46 | 11.43 | 41 | 13.63 | 6.98 |
| 3-month follow-up | 75 | 17.55 | 12.63 | 74 | 10.24 | 6.74 |
| Surf | 43 | 15.63 | 11.79 | 43 | 8.88 | 6.22 |
| Hike | 32 | 20.13 | 13.45 | 31 | 12.13 | 7.08 |

*Note.* MADRS = Montgomery-Åsberg Depression Rating Scale; PHQ-9 = 9-item Patient Health Questionnaire.


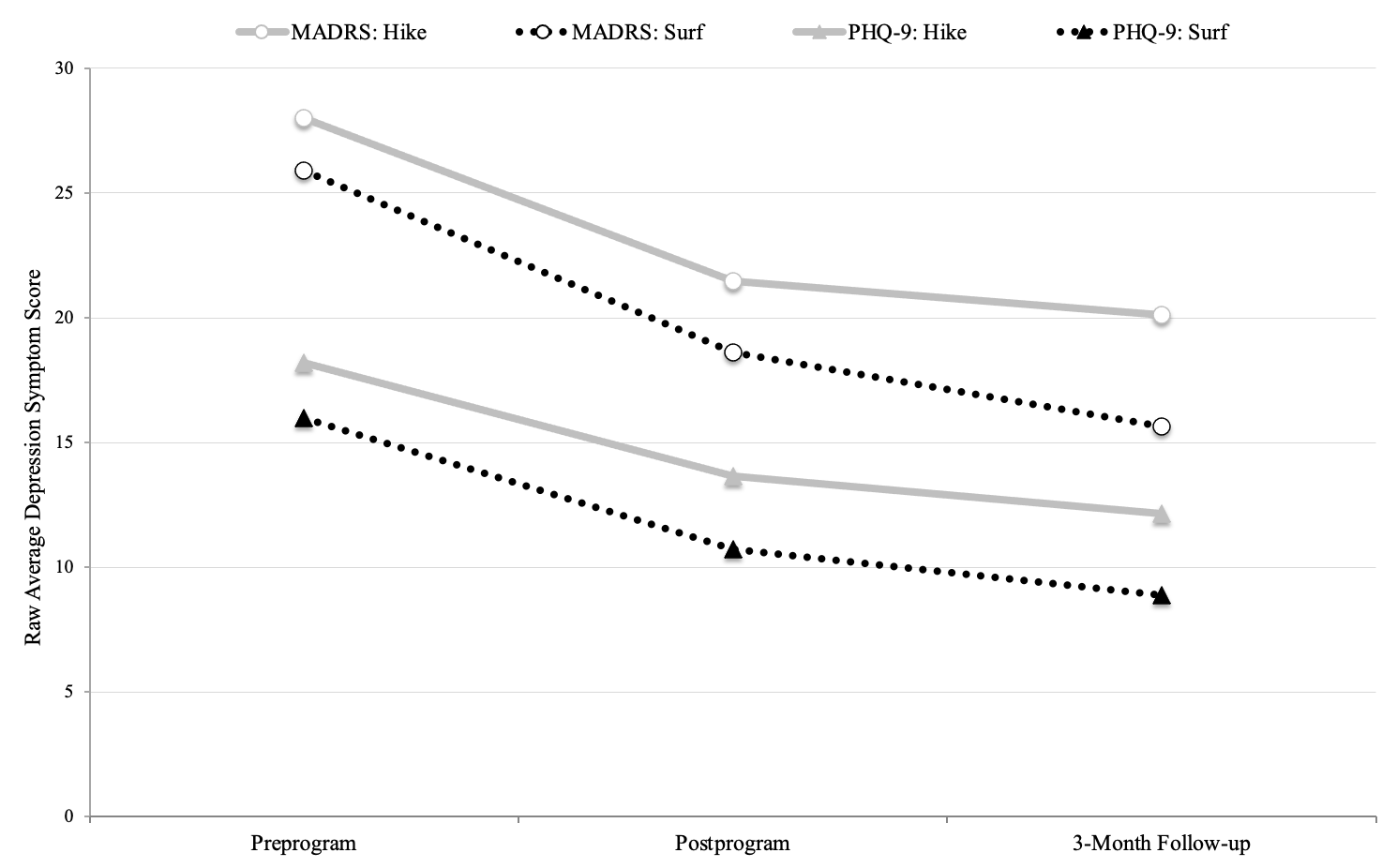


**Fig. A1.** Raw average depression symptom scores at study assessment timepoints. MADRS = Montgomery-Åsberg Depression Rating Scale; PHQ-9 = 9-item Patient Health Questionnaire.

**Table A2**

Means and standard deviations of depression/anxiety symptoms at session assessment timepoints.

|  | | PHQ-4 | | | | | | | | |
| --- | --- | --- | --- | --- | --- | --- | --- | --- | --- | --- |
|  | | Total sample | | | Surf | | | Hike | | |
| Timepoint | | *n* | *M* | *SD* | *N* | *M* | *SD* | *n* | *M* | *SD* |
| Session 1 | Presession 1 | 87 | 6.31 | 2.74 | 44 | 5.55 | 2.56 | 43 | 7.09 | 2.72 |
|  | Postsession 1 | 85 | 3.15 | 2.47 | 44 | 2.50 | 2.24 | 41 | 3.85 | 2.55 |
| Session 2 | Presession 2 | 82 | 6.11 | 2.78 | 44 | 6.11 | 2.94 | 38 | 6.11 | 2.62 |
|  | Postsession 2 | 81 | 2.84 | 2.71 | 43 | 2.51 | 2.59 | 38 | 3.21 | 2.84 |
| Session 3 | Presession 3 | 78 | 5.72 | 3.06 | 42 | 5.43 | 3.22 | 36 | 6.06 | 2.87 |
|  | Postsession 3 | 76 | 2.46 | 2.51 | 40 | 1.88 | 2.14 | 36 | 3.11 | 2.76 |
| Session 4 | Presession 4 | 71 | 5.62 | 2.81 | 40 | 5.55 | 2.81 | 31 | 5.71 | 2.85 |
|  | Postsession 4 | 70 | 3.27 | 2.80 | 39 | 3.05 | 2.87 | 31 | 3.55 | 2.74 |
| Session 5 | Presession 5 | 52 | 5.15 | 3.20 | 27 | 4.81 | 3.08 | 25 | 5.52 | 3.36 |
|  | Postsession 5 | 50 | 2.62 | 2.70 | 26 | 2.62 | 2.94 | 24 | 2.63 | 2.46 |
| Session 6 | Presession 6 | 25 | 4.36 | 3.24 | 14 | 3.36 | 3.34 | 11 | 5.64 | 2.73 |
|  | Postsession 6 | 23 | 2.13 | 2.51 | 12 | 1.58 | 2.54 | 11 | 2.73 | 2.45 |

*Note.* PHQ-4 = 4-item Patient Health Questionnaire.
